# Surgical Outcomes of the Boat-Form Vein Cuff Technique in Peripheral Artery Bypass Grafting

**DOI:** 10.3400/avd.oa.24-00134

**Published:** 2025-05-30

**Authors:** Shun-Ichiro Sakamoto, Anna Tsuji, Motohiro Maeda, Atsushi Hiromoto, Kenji Suzuki, Jiro Honda, Yosuke Ishii

**Affiliations:** 1 Department of Cardiovascular Surgery, Nippon Medical School Musashikosugi Hospital, Kawasaki, Kanagawa, Japan; 2 Department of Cardiovascular Surgery, Nakagami Hospital, Okinawa, Okinawa, Japan; 3 Department of Cardiovascular Surgery, Nippon Medical School, Tokyo, Japan

**Keywords:** vein cuff, peripheral artery bypass, emergency surgery, mesenteric ischemia

## Abstract

**Objectives:** The venous cuff technique has been used primarily for arterial bypass using artificial grafts to the lower extremities. The boat-form vein cuff was designed to allow adjustment of the size and angle of the anastomosis at any anatomic site. We report our experience and outcomes of the original vein cuff technique in various peripheral artery bypass grafting procedures.

**Methods:** A total of 10 patients underwent arterial bypass grafting using a polytetrafluoroethylene (PTFE) graft with a boat-form venous cuff. The indications for the surgery consisted of peripheral artery disease (n = 4), acute limb ischemia (n = 4), chronic mesenteric ischemia (n = 1), and traumatic upper limb ischemia (n = 1). Five patients required emergency surgery. Surgical outcomes, such as mortality and morbidity, limb salvage rate, and graft patency, were examined using perioperative and postoperative follow-up data.

**Results:** There were no operative deaths or serious complications, including amputation of the lower extremity. During the follow-up period (44 ± 36.9 months), the PTFE graft remained patent in 9 patients (90%). In 1 patient, occlusion of the femoropopliteal bypass graft was observed 3 months after surgery.

**Conclusions:** The simple design and creation of the boat-form vein cuff are useful at any anatomical site in peripheral artery bypass grafting with a PTFE graft.

## Introduction

More than 40 years have passed since the venous cuff technique was introduced by Miller. Since then, several variations of the technique have been reported.^1–5)^ Bypass grafting to peripheral arteries with a prosthetic graft using a vein cuff is often used in cases where autologous vein grafts cannot be used, the diameter of the anastomotic vessel is small, or the wall properties of the anastomotic vessel are poor. Advantages of the venous cuff technique include avoiding direct anastomosis between a prosthetic graft and a vessel with an atherosclerotic lesion, forming a large anastomotic opening, and preventing turbulent flow and intimal hyperplasia by increasing the shear stress on the toe and heel of the anastomotic site through the cuff-induced central vortex.^6,7)^ On the other hand, conventional venous cuffs use collar sutures or partial patch formation to create a defined anastomotic opening, making it difficult to adjust the shape and length of the anastomotic opening when suturing an artificial blood vessel. We have previously reported on the boat-form vein cuff technique, in which a vein anastomosed to an artery using the side-to-side technique is trimmed into a boat shape.^8)^ The boat-form technique is easy to perform, has the advantage that the anastomotic opening and angle of the graft and vein can be adjusted as needed, and is expected to be suitable for peripheral vascular surgery at all anatomic sites. Herein, we aimed to report a single center’s experience with the boat-form vein cuff and the surgical outcomes.

This study was performed in accordance with the guidelines of the Declaration of Helsinki and was approved by the Ethics Committee of Nippon Medical School Musashikosugi Hospital in Kanagawa, Japan (No. 734-5-45, 2023).^9)^

## Materials and Methods

A total of 10 patients have undergone peripheral vascular surgery using a polytetrafluoroethylene (PTFE) graft with a boat-form vein cuff in the 9 years since the boat-form vein cuff technique was first used for a femoropopliteal (F-P) artery bypass in 2014.^1)^ The mean age was 71 ± 10.1 years, and 7 (70%) were male. All patients had hyperlipidemia as a risk factor for atherosclerosis, and 3 were current smokers. Eight patients had Trans-Atlantic Inter-Society Consensus Class D aortoiliac or F-P occlusive disease,^10)^ and 4 of them required emergency surgery for the development of acute limb ischemia.

It is the institutional policy to use autologous veins, primarily the great saphenous vein, as the first choice for arterial bypass surgery, but PTFE grafts were used in patients for whom appropriate autologous veins could not be obtained, such as those with varicose veins in the lower extremities or when the great saphenous vein had already been used in coronary artery bypass surgery. PTFE grafts were also used in patients in whom an autologous vein could be obtained, but who had anatomic obstruction factors such as anastomotic compression or graft flexion, or who required emergency revascularization due to acute limb ischemia. The venous cuff technique was used only when the diameter of the peripheral artery was significantly smaller than the PTFE graft and an atherosclerotic lesion was observed grossly at the anastomotic opening.

**Figure 1:**
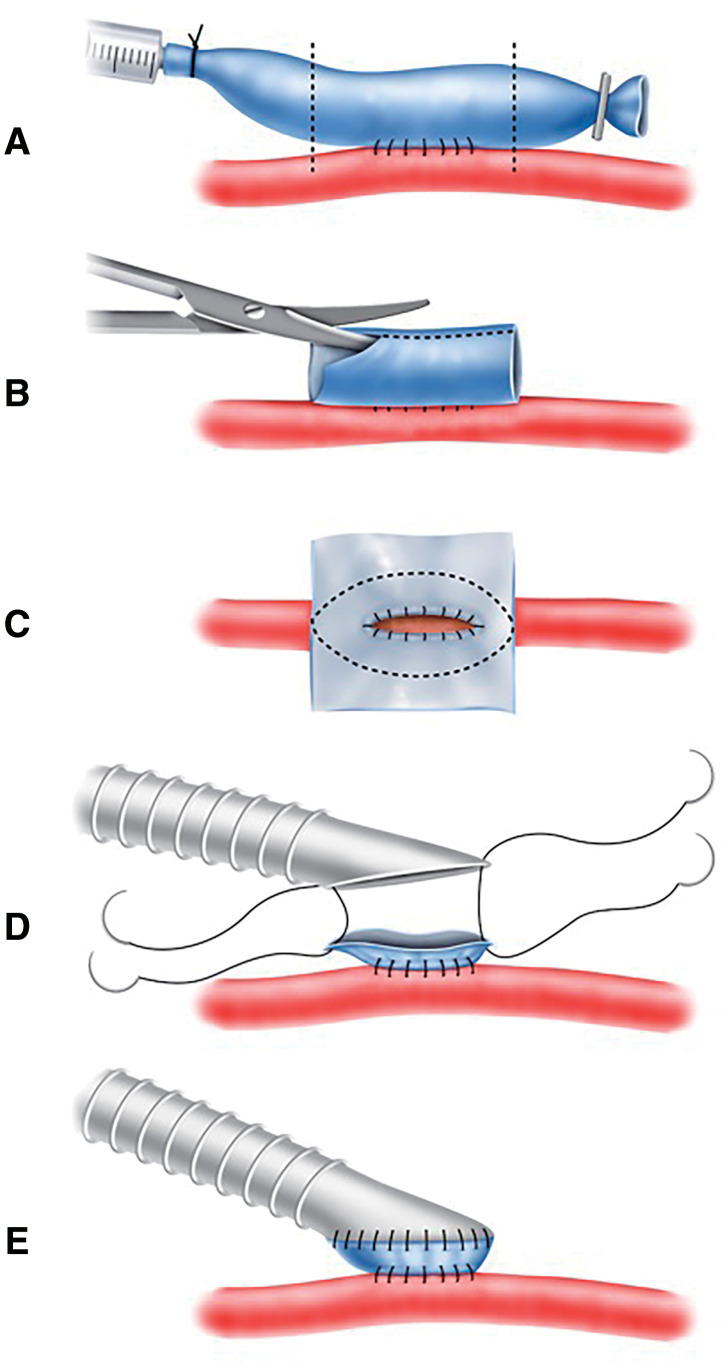
Fig. 1 Illustration of the creation of a boat-form vein cuff. (**A**) Side-to-side anastomosis between the vein and artery. The dotted line indicates the resection line of the vein. (**B**) Longitudinal incision of the vein. (**C**) Trimming of the vein in accordance with the anastomosis diameter and angle of the PTFE graft. (**D**) Sutures are applied to both ends of the PTFE graft and vein cuff. (**E**) Completed boat-form vein cuff anastomosis. PTFE: polytetrafluoroethylene

In the first 5 patients in our study, a 7-cm vein was harvested and the dilated vein was marked in a boat form with a pen and then trimmed.^1)^ However, more recently, the vein was separated at both ends, followed by a longitudinal incision to expose the intimal surface. Thereafter, the vein was trimmed to match the anastomotic design of the PTFE graft (**Fig. 1**). In this case group, a 6-mm PTFE graft was used for all patients, with the length of the arteriotomy incision set at 12 mm and the length of the PTFE anastomosis adjusted to 15–20 mm on the heel side. The vein cuff was created using the great saphenous vein in 3 patients and the collateral saphenous vein in 7 patients. Emergency surgery was performed for acute lower limb ischemia in 4 patients and for traumatic upper limb ischemia due to an axillary laceration in 1 patient. The vein cuff was also used in a superior mesenteric artery bypass for the treatment of mesenteric ischemia in 1 patient.^11)^ Although multiple procedures were performed during the surgeries of 6 patients, the vein cuff was created for a distal anastomosis and a 6-mm PTFE graft was used for the peripheral arterial bypass in all patients.

All patients were administered an antiplatelet agent, and warfarin was used only for the 1st year after the operation with a target prothrombin time-international normalized ratio of 1.5–2. The patients were followed up regularly in the outpatient clinic after surgery. The first contrast-enhanced computed tomography (CT) was obtained 6 and 12 months after surgery. Thereafter, if the patient did not exhibit any ischemic symptoms, the ankle–brachial index and/or CT was obtained once a year to confirm graft patency.

## Results

**Figure 2:**
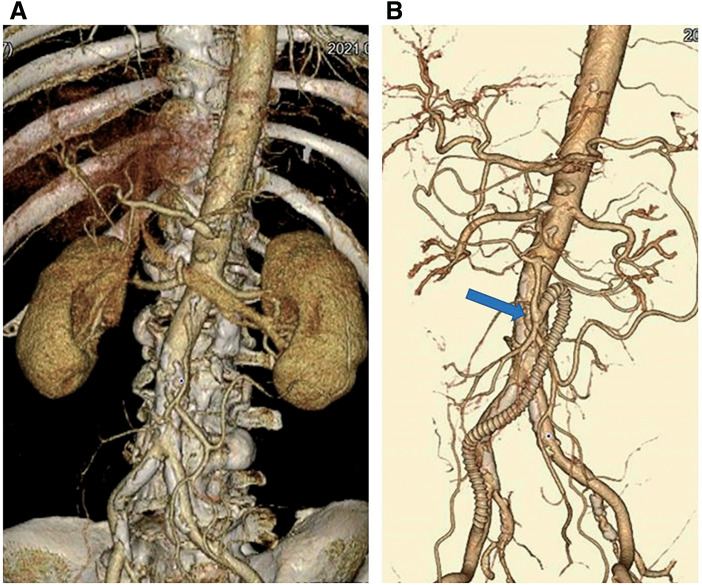
Fig. 2 Superior mesenteric artery bypass for the treatment of chronic mesenteric ischemia (Patient 3). (**A**) 3D reconstruction of preoperatively obtained CT. (**B**) 3D reconstruction of postoperatively obtained CT showing an intact graft and SMA. The blue arrow indicates the site of the vein cuff. 3D: three-dimensional; CT: computed tomography; SMA: superior mesenteric artery

**Figure 3:**
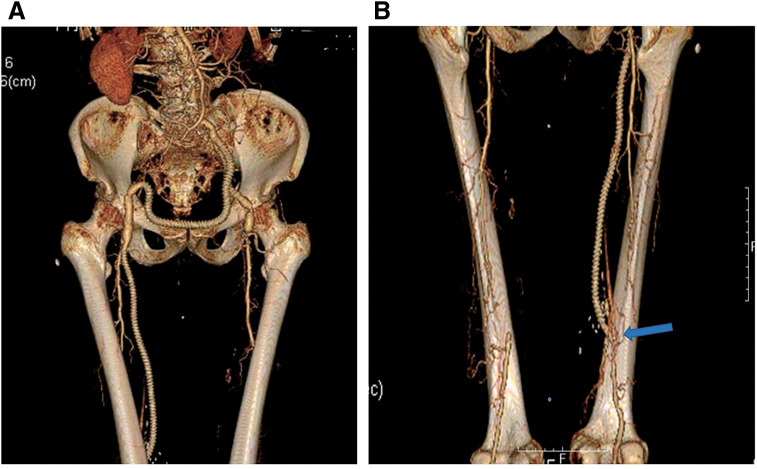
Fig. 3 An emergency case of above knee F-P bypass and multiple inflow procedures for the treatment of acute limb ischemia (Patient 5). (**A**) 3D reconstruction of postoperatively obtained CT showing a patent PTFE graft for a left EIA-CFA bypass and left CFA-right CFA bypass. (**B**) 3D reconstructed posterior view of a PTFE graft above the right knee for an F-P bypass. The blue arrow indicates the site of the vein cuff. 3D: three-dimensional; CFA: common femoral artery; CT: computed tomography; EIA: external iliac artery; F-P: femoropopliteal; PTFE: polytetrafluoroethylene

**Figure 4:**
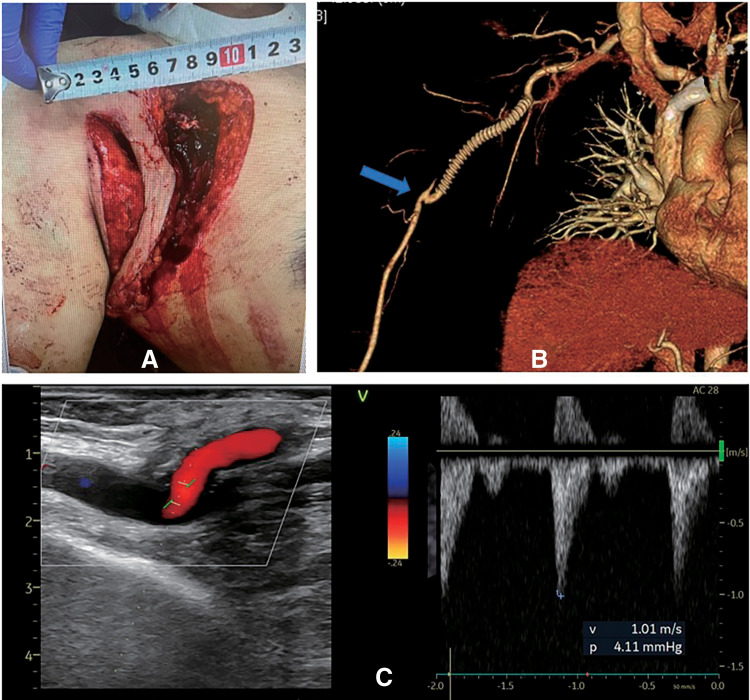
Fig. 4 A case of axillobrachial artery bypass for traumatic obstruction of the brachial artery (Patient 10). (**A**) A 59-year-old man fell from a height, resulting in an axillary laceration and an arteriovenous injury. Axillobrachial artery bypass was performed using a 6-mm PTFE graft. (**B**) 3D reconstruction of postoperatively obtained computed tomography image showing the site of the vein cuff (blue arrow). (**C**) Color Doppler of the vein cuff showing a patent graft, absence of significant stenosis, and high-speed turbulent flow. PTFE: polytetrafluoroethylene

**Table 1** shows the patients' characteristics and vascular surgeries in which the boat-form vein cuff technique was used. The mean operative time was 289 ± 102 minutes. The treatment of chronic mesenteric ischemia (Patient 3) required multiple procedures, including ilio-superior mesenteric artery bypass with a venous cuff, division of the median arcuate ligament, and angioplasty of the celiac artery, which were performed in collaboration with physicians from other departments (**Fig. 2**). The most time-consuming surgery was performed in Patient 5, in whom 3 bypasses were created from the inflow site to the peripheral anastomosis, in addition to an endarterectomy of the external iliac and common femoral arteries. The operative time was 555 minutes (**Fig. 3**). Among the emergency surgeries for acute ischemia of the lower extremity, 2-compartment fasciotomy was performed in 3 patients (Patients 5, 6, and 7) for acute compartment syndrome that developed due to postoperative revascularization. In Patient 6, a postoperative CT revealed local necrosis of the gastrocnemius muscle, and the mean blood creatine phosphokinase (CPK) level was 3216 ± 4387.8. The CPK level was the highest in Patient 7, with a maximum CPK value of 9000 U/L immediately after surgery. Thus, continuous hemodialysis was performed for 4 days. Skin grafting was performed to the relaxed skin tension lines on postoperative day 15 in Patient 7. In the patient with a traumatic brachial artery injury (Patient 10), the artery was initially reconstructed in the emergency room using an autologous vein. However, after closure of the wound, the graft became bent and occluded. Thus, the patient was immediately transferred to the operating room for bypass using a PTFE graft (**Fig. 4**).

**Table:** Table 1 Patients’ characteristics

Case	Sex	Age	Diseases	Elective or emergency	Procedures
1	M	70	PAD	Elective	BK F-P bypass
2	M	75	PAD	Elective	BK F-P bypass
3	M	66	CMI	Elective	Division of MAL, CIA-SMA bypass
4	M	66	PAD	Elective	EA of Lt EIA and PA, Lt BK F-P bypass
5	M	81	ALI	Emergent	Lt CIA-EIA bypass, EA of Lt EIA, Lt EIA-Rt CFA bypass, EA of Rt CFA, Rt AK F-P bypass
6	F	64	ALI	Emergent	Rt EIA-Lt EIA bypass, EA of Lt CFA, Lt AK F-P bypass
7	F	90	ALI	Emergent	Lt EIA-Rt CFA bypass, Rt BK F-P bypass
8	F	77	ALI	Emergent	BK F-P bypass, thrombectomy
9	M	73	PAD	Elective	BK F-P bypass
10	M	59	Trauma	Emergent	Axillobrachial artery bypass

ALI: acute limb ischemia; BK: below-knee; CIA: common iliac artery; CMI: chronic mesenteric ischemia; EA: endarterectomy; EIA: external iliac artery; F: female; F-P: femoropopliteal; Lt: left; M: male; MAL: median arcuate ligament; PAD: peripheral arterial disease; Rt: right; SMA: superior mesenteric artery

There were no operative deaths or serious complications, such as amputation of the upper or lower extremities, in our study. Postoperative contrast-enhanced CT showed that the graft was patent in all patients. All upper and lower limbs were preserved, and 5 patients were transferred to postoperative rehabilitation facilities. During the postoperative follow-up period (44 ± 36.9 months), the PTFE graft remained patent in 9 patients (90%). One patient (Patient 3) developed occlusion of the F-P bypass graft at 3 months postoperatively.

## Discussion

**Figure 5:**
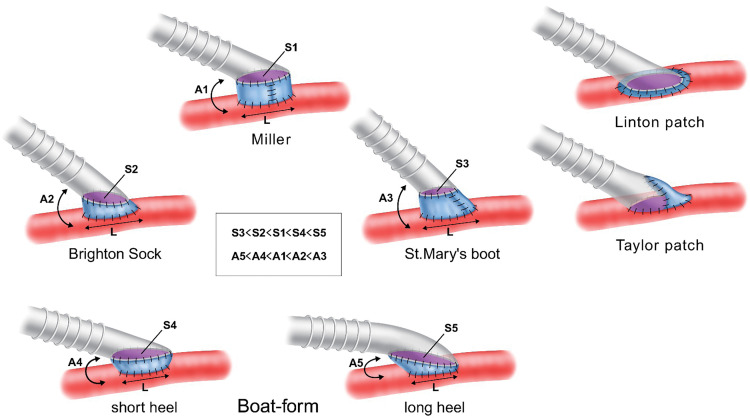
Fig. 5 Vein cuff and venous patch techniques. The anastomotic size of the vein cuff at the arterial incision length is the largest in the boat-form vein cuff and the smallest in the St. Mary’s boot vein. However, the anastomotic angle of a prosthetic graft with vein cuff decreases as the anastomosis size increases. The boat-form vein cuff has the largest angle among the 4 vein cuffs. The boat-form vein cuff differs from other vein cuffs in that the anastomosis size and angle can be adjusted by changing the length of the heel portion of the cuff. A: anastomotic angle between prosthetic graft and target artery; L: length of arteriotomy; S: anastomotic size of prosthetic graft

Artificial grafts are inferior to autologous veins in terms of patency of the bypass to the popliteal artery.^12)^ To overcome this challenge, Miller developed the venous cuff technique, in which a vein is inserted between the graft and the artery. Randomized studies have demonstrated that the venous cuff technique may improve graft patency.^13,14)^ However, the creation of collared venous cuffs, such as the Miller cuff and Saint Mary’s boot, is not technically easy and can result in cross sutures between the longitudinal suture of the cuff and the arterial wall or PTFE graft.^1,2)^ The venous cuff using the Brighton method and the venous patch using the Linton method do not include the longitudinal suture, which is a drawback of the collared cuff. However, the size of the anastomosis is determined by the length of the arteriotomy incision, so the size cannot be adjusted according to the suture angle of the PTFE graft.^3,4)^ The Taylor method requires direct suturing of the graft to the artery, reducing the benefits of the cuff.^5)^ The boat-form vein cuff has the largest anastomosis size per length of arteriotomy when compared with the other vein cuff methods. Furthermore, the graft can be freely trimmed, and the anastomotic angle can be adjusted by changing the cuff’s shape (**Fig. 5**). The hemostatic state of the cuff can be easily checked without crossing sutures during cuff creation. Thus, the cuff can be created according to the surgical field, and peripheral vascular surgery can be easily performed at any anatomical site.

In our study, PTFE grafts were used in 4 emergency operations despite the availability of vein grafts. These grafts may have been used to reduce the time spent harvesting autologous veins and because the interposition of a vein cuff produces better results than PTFE alone when bypassing the popliteal artery below the knee.^15)^ Furthermore, the boat-form vein cuff is easy to create, especially in patients in whom the inflow procedure requires time. For example, in Patients 5 and 6, it was possible to simultaneously create the vein cuff and perform inflow procedures by securing a separate operative field. All emergency surgeries, including those performed in Patients 5 and 6, were performed for Rutherford Class IIB acute limb ischemia that developed due to native artery occlusion and critical limb ischemia.^16)^ Although postoperative reperfusion injury developed in 3 of these patients (75%), limb salvage was achieved in all 4 patients. Guntani et al. reported a high limb salvage rate despite a low long-term patency rate in their study.^17)^ They speculated that the high limb salvage rate may be due to an adequate supply of direct blood flow. In our study as well, the boat-form cuff technique may have been useful in terms of time-to-reperfusion and ensuring blood flow.

In the present study, PTFE bypass with a boat-form vein cuff was performed to the brachial artery and superior mesenteric artery (SMA) in addition to revascularization of the lower extremity. Direct anastomosis of the prosthetic graft to these arteries was avoided only because of a caliber mismatch with the PTFE. PTFE graft bypass to the brachial artery has been reported in several cases, primarily for the treatment of brachial artery aneurysms.^18)^ In a review of 49 patients with this condition, the PTFE graft was used in 9 patients (18%), and all peripheral anastomoses were direct anastomoses without a venous cuff. In the upper arm, where subcutaneous fat is limited, autologous veins are usually used to avoid infection. In Patient 10 in our study, venous grafting would have been preferred due to the trauma and contamination. However, due to the long graft length and the compression caused by the residual subcutaneous hematoma, we had to use a PTFE graft. The PTFE graft is frequently used for SMA bypass in chronic mesenteric ischemia because a 4-mm or longer autologous vein is required, which may kink depending on the bypass length.^19)^ Furthermore, the incidence of graft failure in the early postoperative period is reported to be less than half that of autologous venous bypass.^20)^ In Patient 3 in our study, we performed a C-loop retrograde PTFE graft bypass with iliac artery inflow. Although several similar revascularization procedures have been reported, none have used a venous cuff for mesenteric artery bypass.^21)^ Because of the good clinical course and graft blood flow after revascularization of both brachial and mesenteric arteries, PTFE bypass using a boat-form vein cuff may be used for all peripheral arterial revascularization procedures, not only for arterial diseases of the lower extremities.

This study had some limitations. Because autologous veins or PTFE grafts are always used alone as grafts in peripheral artery bypass surgery, venous cuffs are used in very few cases. Furthermore, the study was conducted at a single institution. To overcome these limitations, further multicenter studies are required to compare the outcomes of treatment with and without the use of the boat-form vein cuff.

The boat-form vein cuff has a large anastomotic opening due to the outwardly warped cuff edge, which facilitates anastomosis with the PTFE graft. Thus, it may be useful in cases where the operative field is deep and narrow. On the other hand, the results of the present study do not indicate that the boat-form vein cuff is superior to other vein cuffs. Previous studies have used computer models to analyze the anastomotic blood flow patterns and wall shear stress distribution in end-to-end anastomosis using venous cuffs.^22,23)^ In the future, similar analyses will be required to compare the blood flow pattern in a boat-form vein cuff, a Miller cuff, other vein cuffs, and precuffed PTFE grafts.^24,25)^

## Conclusion

The simple design and creation of the boat-form vein cuff are useful at any anatomical site in peripheral artery bypass grafting with a PTFE graft.

## Declarations

### Acknowledgments

We acknowledge the excellent assistance of Masako Izumi in preparing the manuscript.

### Disclosure statement

The authors do not have any conflicts of interest to declare.

### Author contributions

Study conception: SS, JH

Data collection: SS, MM, AT, AH

Analysis: SS

Investigation: MM, SS, KS, JH

Funding acquisition: none

Manuscript preparation: SS, MM, YI

Critical review and revision: all authors

Final approval of the article: all authors

Accountability for all aspects of the work: all authors.
